# Discovery of a Generalization Gap of Convolutional Neural Networks on COVID-19 X-Rays Classification

**DOI:** 10.1109/ACCESS.2021.3079716

**Published:** 2021-05-13

**Authors:** KAOUTAR BEN AHMED, GREGORY M. GOLDGOF, RAHUL PAUL, DMITRY B. GOLDGOF, LAWRENCE O. HALL

**Affiliations:** 1 Department of Computer Science and Engineering, University of South Florida, Tampa, FL 33620, USA; 2 Department of Laboratory Medicine, The University of California, San Francisco, CA 94143, USA; 3 Department of Radiation Oncology, Massachusetts General Hospital, Boston, MA 02115, USA; 4 Department of Radiation Oncology, Harvard Medical School, Boston, MA 02115, USA

**Keywords:** Coronavirus (COVID-19), pneumonia, chest X-ray images, deep learning, confounder

## Abstract

A number of recent papers have shown experimental evidence that suggests it is possible to build highly accurate deep neural network models to detect COVID-19 from chest X-ray images. In this paper, we show that good generalization to unseen sources has not been achieved. Experiments with richer data sets than have previously been used show models have high accuracy on seen sources, but poor accuracy on unseen sources. The reason for the disparity is that the convolutional neural network model, which learns features, can focus on differences in X-ray machines or in positioning within the machines, for example. Any feature that a person would clearly rule out is called a confounding feature. Some of the models were trained on COVID-19 image data taken from publications, which may be different than raw images. Some data sets were of pediatric cases with pneumonia where COVID-19 chest X-rays are almost exclusively from adults, so lung size becomes a spurious feature that can be exploited. In this work, we have eliminated many confounding features by working with as close to raw data as possible. Still, deep learned models may leverage source specific confounders to differentiate COVID-19 from pneumonia preventing generalizing to new data sources (i.e. external sites). Our models have achieved an AUC of 1.00 on seen data sources but in the worst case only scored an AUC of 0.38 on unseen ones. This indicates that such models need further assessment/development before they can be broadly clinically deployed. An example of fine-tuning to improve performance at a new site is given.

## I. INTRODUCTION

At the end of the year 2019, we witnessed the start of the ongoing global pandemic caused by Coronavirus disease (COVID-19) which was first identified in December 2019 in Wuhan, China. As of December 2020, more than 75 million cases are confirmed with more than 1.67 million confirmed deaths worldwide [1]. In the first few months of the pandemic, the testing ability was limited in the US and other countries. Testing for COVID-19 has been unable to keep up with the demand at times and some tests require significant time to produce results (days) [2]. Therefore, other timely approaches to diagnosis were worthy of investigation [3]. Chest X-rays (CXR) can be used to give relatively immediate diagnostic information. X-ray machines are available in almost all diagnostic medical settings, image acquisition is fast and relatively low cost.

**FIGURE 1.**
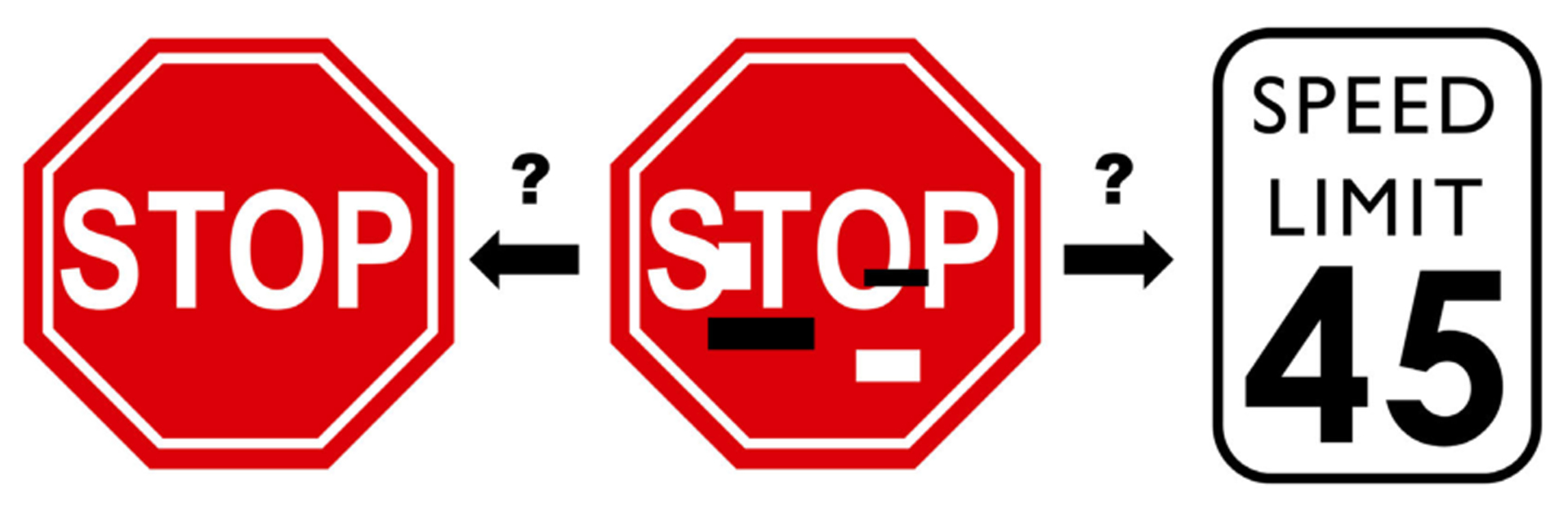
Modified Stop sign could be classified in a dangerous way.

Multiple studies have been published claiming the possibility of diagnosing COVID-19 from chest X-rays using machine learning models with very high accuracy. However, we show that these models will likely generalize to unseen data sources very poorly because they likely have learned spurious (confounding) features instead of true and relevant COVID-19 radiographic markers. These studies rely on deep learning approaches using convolutional neural networks (CNN) which automatically extract features. A great concern with deep neural networks is whether the features they have learned for a particular problem are relevant. As an example, a study has shown that a CNN which learned to identify traffic signs will misclassify a stop sign as a 45 mile per hour speed limit sign, if just a couple of strips are placed on the sign without obscuring any text. This was demonstrated by the addition of a black or white sticker that did not obscure the ‘STOP’ word on the sign, a change that would have no effect on the human interpretation of the sign [4]. Fig 1 shows an example that we would all interpret as a stop sign, but a CNN might misclassify.

Recent surveys [5], [6] have discussed multiple papers applying Artificial Intelligence (AI) to CXR imaging of COVID-19 and reporting very high performance results. In Table 1, we review these papers, in addition to others. We added a column to report the testing data splits used for the evaluation of their method(s). As seen in Table 1, in most of those papers authors used subsets of train/validation/test from the same data source, others opted for a cross validation evaluation method, which also mixed train/validation/test sources. In this paper, we show how the use of the same sources in train/test sets leads to the high accuracy that these models have achieved. In addition, the majority of these papers used low quality images, some are extracted from PDF files of scientific publications. This approach increases the likelihood of introducing image processing artifacts, which further increases the risk of learning confounders rather than real pathologic features.

Additionally, in some studies, [18], [28], [29], [31], [32], [35], [39]–[44] the pneumonia/normal class dataset was based on a pediatric dataset (age of patients 1–5 years of age). Whereas, the average age of the COVID-19 class was *>*40 years. By looking at the pneumonia image, it is evident that the sizes of the rib cages and thoracic structures of the pneumonia dataset are different from the COVID-19 cases, due to the age difference. Since convolutional neural networks have been shown to be able to learn the concept of size [45] (e.g. lung size), these models were likely capturing age-related features to differentiate pneumonia/normal cases and COVID-19 cases, as a proxy for age rather than pathologic diagnosis.

In contrast, findings in [36]–[38] support our observations where deep learning models perform very well on seen sources and poorly on unseen ones. Furthermore, The authors in [36] investigated and showed, using saliency maps and generative adversarial networks (GANs), that the model is actually learning medically irrelevant features to differentiate between labels instead of COVID-19 pathology. This work essentially demonstrated that the deep learning algorithms were looking at non-lung regions of the chest X-ray to classify the majority of images.

Furthermore, more recent studies [46] performing meta-analysis of papers suggesting AI methods for COVID-19 detection have started to appear. Authors in [46] questioned the clinical utility of the reviewed papers and discussed their methodological flaws.

The focus of this paper is to determine whether deep learning models can be considered reliable for diagnosing COVID-19 based on reasonable biomarkers, or are they only learning shortcuts (confounders) to differentiate between classes. To evaluate this question, we worked with 655 chest X-rays of patients diagnosed with COVID-19 and a set of 1,069 chest X-rays of patients diagnosed with other pneumonia that predates the emergence of COVID-19.

## II. MATERIALS AND METHODS

### A. DATASETS

In our previous work [24], we used COVID-19 images from three main sources [47], [48] and [49]. Note that these sources were and still are largely used in the majority of research papers related to the prediction of COVID-19 from X-rays. We later identified a number of potential problems with these sources. Many of these images are extracted from PDF paper publications, are pre-processed with unknown methods, down-sampled, and are 3 channel (color). The exact source of the image is not always known and the stage of the disease is unknown.

For the COVID-19 class, three sources were used in this work, BIMCV-COVID-19+ (Spain) [50], COVID-19-AR (USA) [51] and V2-COV19-NII (Germany) [52]. For readability, we will label each dataset both by its name and also its country of origin, since the names of each dataset are similar and may confuse the reader. (i) BIMCV COVID-19+ (Spain) is a large dataset from the Valencian Region Medical ImageBank (BIMCV) containing chest X-ray images CXR (CR, DX) and computed tomography (CT) imaging of COVID-19+ (positive) patients along with their radiological findings and locations, pathologies, radiological reports (in Spanish) and other data. The images provided are 16bits in png format. (ii) COVID-19-AR (USA) is a collection of radiographic (X-ray) and CT imaging studies of patients from The University of Arkansas for Medical Sciences Translational Research Institute who tested positive for COVID-19. Each patient is described by a limited set of clinical data that includes demographics, comorbidities, selected lab data and key radiology findings. The provided images are in DICOM format. (iii) V2-COV19-NII (Germany) is a repository containing image data collected by the Institute for Diagnostic and Interventional Radiology at the Hannover Medical School. It includes a dataset of COVID-19 cases with a focus on X-ray imaging. This includes images with extensive meta-data, such as admission, ICU, laboratory, and anonymized patient data. The set contains raw, unprocessed, gray value image data as Nifti files.

Each patient in the datasets had different X-ray views (Lateral, AP or PA) and had multiple sessions of X-rays to assess the disease progress. Radiology reports and PCR test results were included in both BIMCV COVID-19+ and COVID-19-AR (USA) sources. We selected patients with AP and PA views. After translating and reading all the sessions reports coupled with PCR results, only one session per patient was chosen based on the disease stage. We picked the session with a positive PCR result and most severe stage.

As discussed in [46], using raw data in its original format is recommended. In our study, we included all raw COVID-19 datasets that were available to us (COVID-19-AR (USA) [51] and V2-COV19-NII (Germany) [52]). To avoid creating confounders based on the CXR view, we used frontal view(AP/PA) CXRs in both classes. To assure the validity of the ground truth, we made sure not to rely only on a positive RT-PCR but also on the associated CXR report confirming and supporting the test results.

**FIGURE 2.**
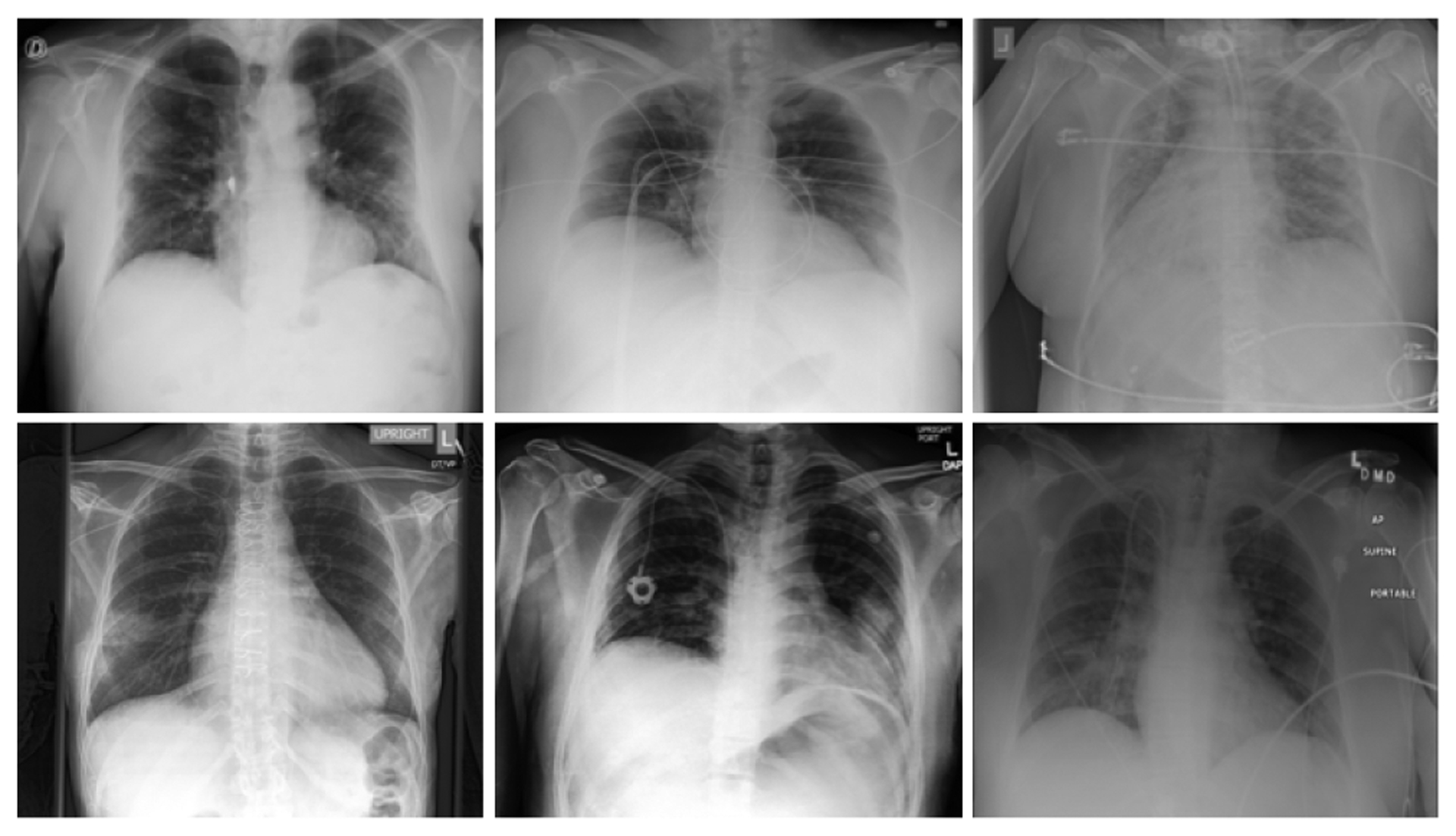
Samples of the input X-rays. TOP: COVID-19 cases. BOTTOM: Pneumonia cases.

For the non-COVID-19 class, pneumonia cases were used because they are expected to be the hardest CXR images to differentiate from COVID-19 and because a use case for deep learned models to detect COVID-19 will be for patients that have some lung involvement. The pneumonia class data came from 3 sources: (i) the National Institute of Health (NIH) dataset [53], (ii) Chexpert dataset [54] and (iii) Padchest dataset [55]. The NIH and Chexpert dataset had pneumonia X-ray images with multiple labels (various lung disease conditions), but for simplicity, we chose the cases that had only one label (pneumonia). Only X-rays with a frontal view (AP or PA) were used in this work. Three samples of COVID-19 and three pneumonia X-ray images are shown in Fig 2.

### B. DATA PRE-PROCESSING

**FIGURE 3.**
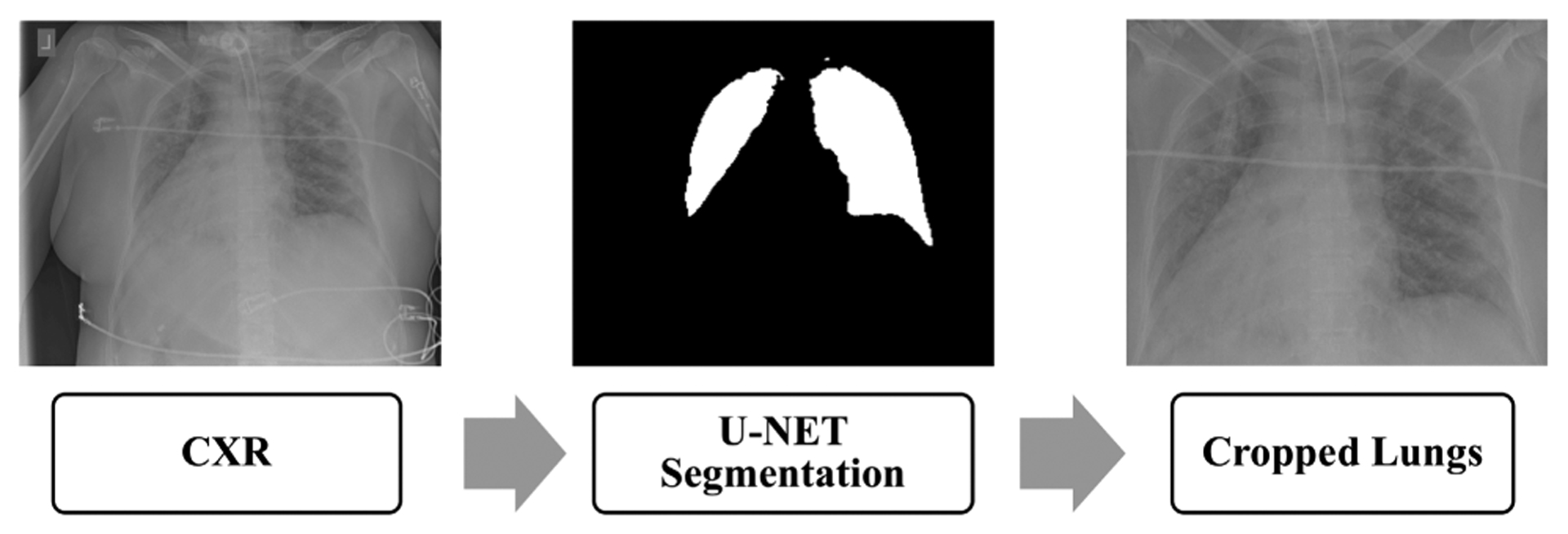
Pipeline of Lung ROI cropping.

As stated in the previous section, the obtained images come in different formats. Padchest [55] and BIMCV-COVID-19+ (Spain) [50] datasets were processed by rescaling the dynamic range using the DICOM window width and center, when available. We do not know of any pre-processing steps applied to the other datasets. As a first step we normalized all the images to 8 bits PNG format in the [0– 255] range. The images were originally 1 grayscale channel, we duplicated them to 3 channels for use with pre-trained deep neural networks. The reason behind this is that Resnet50, the model that we utilized as a base model was pretrained on 8 bit color images. In order to reduce the bias that might be introduced by the noise present around the corners of the images (dates, letters, arrows … etc), we automatically segmented the lung field and cropped the lung area based on a generated mask. We used a UNET model pre-trained by [56] on a collection of CXRs with lung masks. The model generates 256 × 256 masks. We adapted their open source code [56] to crop the image to obtain bounding boxes containing the lung area based on the generated masks. We resized the masks to the original input images size. We then added the criteria to reject some of the failed crops based on the generated mask size. If the size of the cropped image is less than half of the size of the original image or if the generated mask is completely blank then we do not include it in the training or test set. Fig 3 illustrates the steps of mask generation and lung ROI cropping.

For data augmentation, 2, 4, −2, and −4 degree rotations were applied and horizontal flipping was done followed by the same set of rotations. By doing so, we generated 10 times (original images, horizontal flipping, 4 sets of rotated images each from original and flipped images) more images than the original data for training. We chose a small rotation angle as X-rays are typically not rotated much.

### C. MODEL TRAINING

In this study, pre-trained ResNet50 [57] was fine-tuned. As a base model, we used the convolutional layers pretrained on ImageNet and removed the fully connected layers of Resnet50. Global Average pooling was applied after the last convolutional layer of the base model and a new dense layer of 64 units with ReLU activation function was added. Then, a Dense layer with 1 output with sigmoid activation was added using dropout with a 0.5 probability. All the layers of the base model were frozen during the fine-tuning procedure except the Batch Normalization layer to update the mean and variance statistics of the new dataset (X-rays). The total number of trainable parameters was 184K, which was helpful for training with a small dataset. The architecture is summarized in Table 2.

The model was fine-tuned using the Adam [58] optimizer for learning with binary-cross-entropy as the loss function and a learning rate of 10^−4^. We set the maximum number of epochs to 200, but we stopped the training process when the validation accuracy did not improve for 5 consecutive epochs. The validation accuracy reaches its highest value of 97% at epoch 100.

## III. EXPERIMENTAL RESULTS AND DISCUSSION

In this section we investigate the robustness and generalization of deep convolutional neural networks (CNNs) in differentiating between COVID-19 positive and negative class (non-COVID-19 pneumonia). For this purpose, we did a baseline experiment similar to what the reviewed papers have conducted. CNN models were trained on 434 COVID-19 and 430 pneumonia chest X-rays images randomly selected from all the sources that we introduced in the previous section. For validation, 40 COVID-19 and 46 pneumonia cases were utilized. We then tested on unseen left-out data of 79 COVID-19 (30 from BIMCV COVID-19+, 10 from COVID-19-AR (USA) and 39 from V2-COV19-NII (Germany) ) cases and 303 pneumonia (51 from NIH and 252 from Chexpert) samples. For comparison purposes, we used another fine-tuning methodology where we unfroze some of the base model convolutional layers. Thus, the weights of these layers get updated during the training process. In particular, we unfroze the last two convolutional layers of Resnet50. We also used the two fine-tuning strategies to train another model with VGG-16 as the base model, pretrained on ImageNet. The testing results are summarized in Table 3.

**FIGURE 4.**
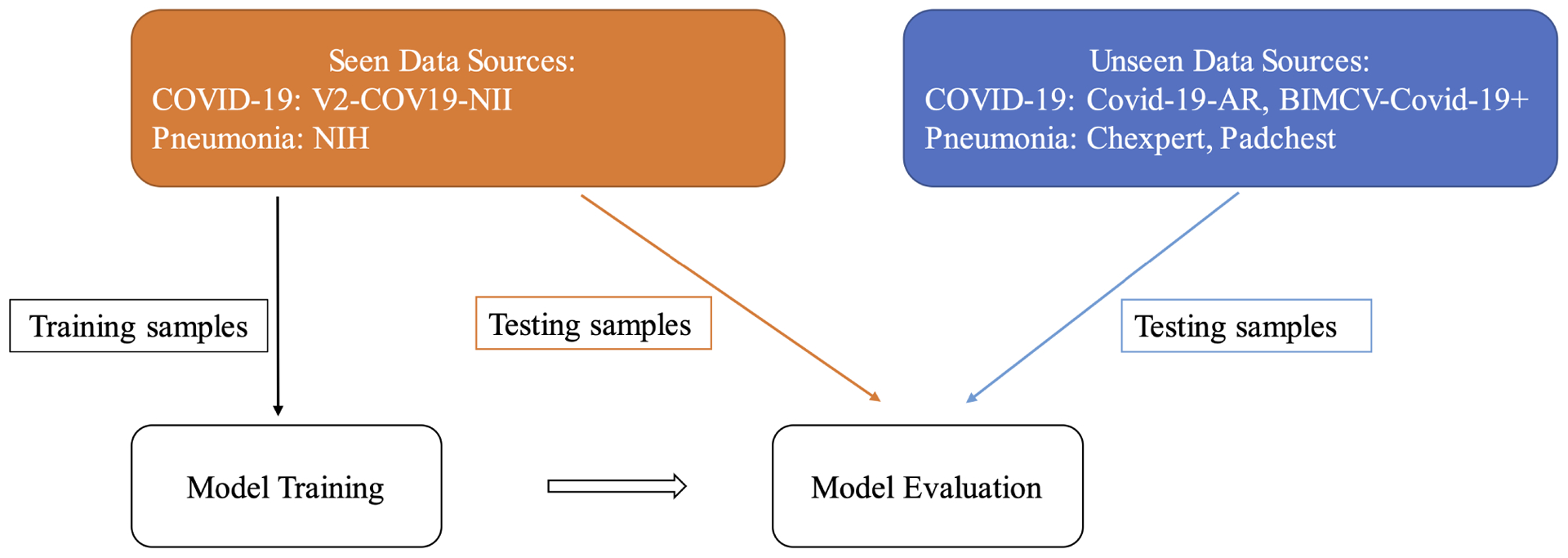
Workflow of the generalization gap experiments. A subset from the training data sources (in orange) is used for model training. Then, we compare model evaluation using 1) a held-out subset from the same training sources (seen) versus 2) using testing samples from unseen data sources (in blue).

As expected, and as seen in Table 3, both models and both fine-tuning methods were able to achieve high performance on an unseen test set from the same sources. In order to investigate the generalization of these models (which is the main focus of this paper), evaluation was performance on external data sources for which there were no examples in the training data. Experiments were done with training data from just one source per class and testing data from sources not used in training ( see Fig. 4 ). The Resnet-50 architecture with the Finetune1 method was used for the rest of the experiments in this paper.

**FIGURE 5.**
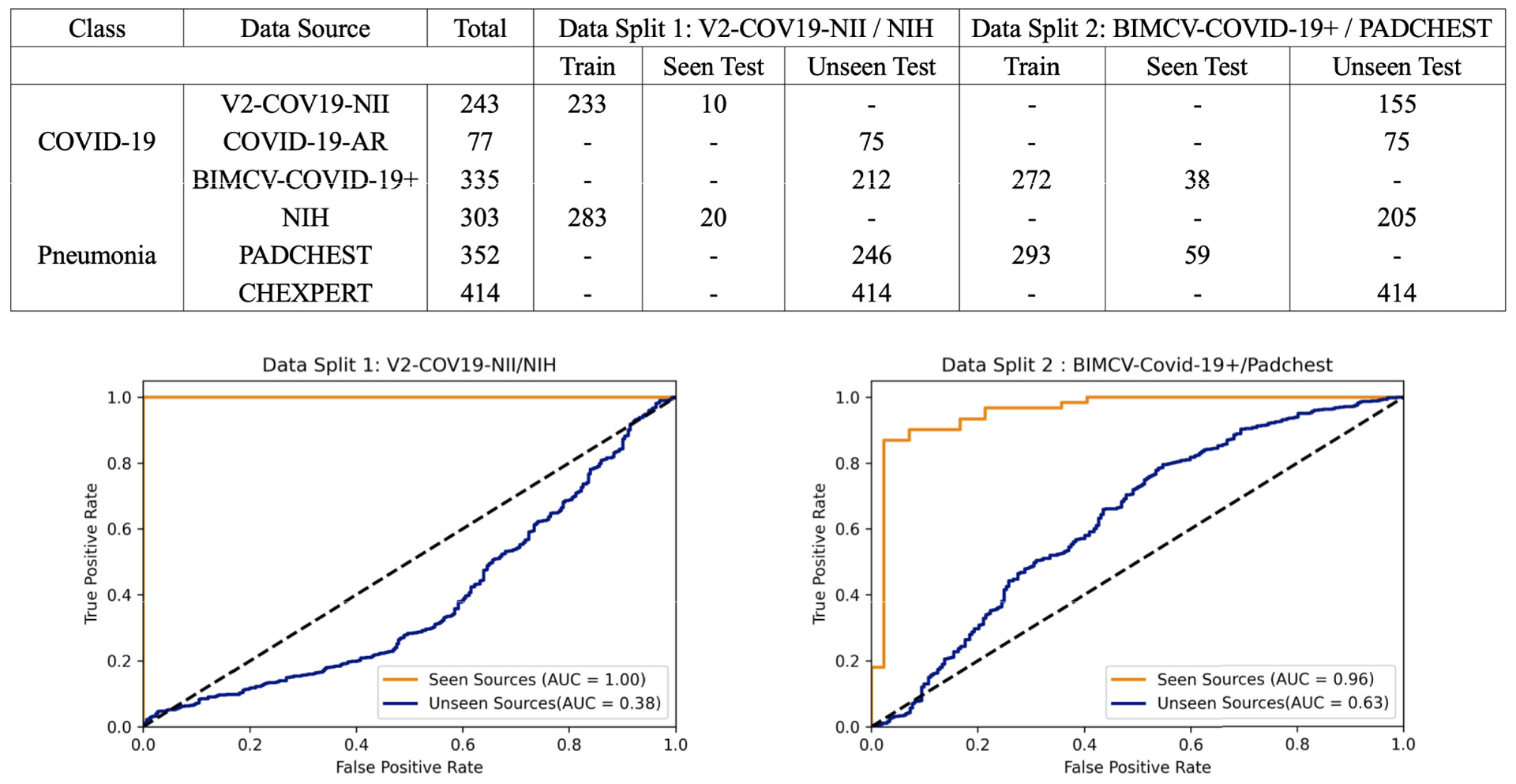
Overview of data splits (top) and comparison of AUC results (bottom) on seen vs. unseen test data sources. Note the high accuracy when held out test data is from a source included in the training set (mixing of train/test data sources). The high accuracy of these models vanishes when the data sources of the training sets are kept strictly separated from the data sources of the test sets.

The data overview table at the top of Fig. 5 shows details of data splits used in our experiments with total number of samples used for training and testing phases. As seen in the table, we first trained the model using the V2-COV19-NII (Germany) data source for the COVID-19 class and NIH for pneumonia (Data Split 1). We then compared the AUC results on a randomly held-out subset from the seen sources (V2-COV19-NII (Germany) and NIH) versus unseen sources.

As seen in the AUC graph in Fig. 5 to the left, the model achieves perfect results (AUC = 1.00) on left-out test samples from seen sources (images from the same dataset source on which the model was trained), but it performs poorly (AUC = 0.38) on images from unseen sources. Using the McNemar’s test [59], we calculated a p-value of 1.78 × 10^−70^ which is way lower than the significance threshold, *alpha* = 0.01. There is a significant difference between the model’s performance on seen vs unseen sources with 99% confidence.

Clearly the model was unable to generalize well to new data sources, which might indicate that the model is relying on confounding information related to the data sources instead of the real underlying pathology of COVID-19. The fact that its performance (AUC=0.38) is less than AUC=0.5 (worse than random), strongly suggests that the model is relying on confounding information. The perfect score on the data from the seen dataset source also hints at confounders, as it is unlikely that any algorithm could perfectly distinguish COVID-19 positive versus pneumonia patients based on lung findings alone. On the other hand, it is highly likely that perfect classification could be performed based on the features related to the images data-source. To give a human analogy, a radiologist would find it easier to classify COVID-19+ versus COVID-19-negative chest X-rays by looking at the year in which the image was taken (pre-2020 versus post), rather than by looking at the image itself.

In an experiment to see if a model built with data from similar sources for the two classes (COVID-19 and Pneumonia) can result in more general models, we chose a second data split (data split 2) with BIMCV-COVID-19+ (Spain) data as the source for COVID-19 and Padchest for Pneumonia. These two sources come from the same regional healthcare system (Valencia, Spain), both were prepared by the same team and underwent the same data pre-processing. We anticipated that reducing the differences between classes in terms of image normalization, hospitals, scanners, image acquisition protocols, etc would enable the model to only concentrate on learning medically-relevant markers of COVID-19 instead of source specific confounders. Details about data split 2 can be found in the data overview table on top of Fig. 5.

The results in the AUC graph in Fig 5 to the right show that the model still exhibits high performance on seen sources but generalizes poorly to external sources. Using the McNemar’s test [59], we calculated a p-value of 5.39 × 10^−82^ which is way lower *alpha* = 0.01. Therefore there is a statistically significant difference between the model’s performance on seen vs unseen sources with 99% confidence.

We can see that even having both classes from the same hospital system did not prevent the model from learning data-source specific confounders. However, in contrast to the model trained on Data Split 1, this model has slightly worse performance on data from seen sources (AUC=0.96 for data split 2 vs AUC=1.00 for data split 1) and better performance on data from unseen sources (AUC=0.63 for data split 2 vs AUC=0.38 for data split 1). Notably, the second model’s performance is better than random (AUC*>*0.5). This suggests that the algorithm may have learned some clinically salient features, although once again, the majority of its performance appears to be based on confounders.

We can also observe that it is possible that confounders found in some data sources can generalize across sources. For example when training using the BIMCV-COVID-19+ (Spain) data source, the model had an accuracy of 88% on COVID-19-AR (USA), which is an unseen source. However when training using V2-COV19-NII (Germany) data source, the model only achieved an accuracy of 68% on this same unseen source (COVID-19-AR (USA)).

As a possible solution, we tried fine-tuning the trained model from the previous experiment (data split 1) using multiple sources for each class, using a subset of 80 samples from BIMCV-COVID-19+ (Spain) for the COVID-19 class and a subset of 80 samples from Chexpert for the pneumonia class. Both these sources were considered unseen in the experiment with data split 1 described in the data overview table on top of Fig. 5. As seen in Table 4, fine-tuning with subsets from unseen sources improves the model’s overall performance on those sources. We hypothesize that fine-tuning helps the model to ignore noisy features and data-source related confounders and instead concentrate on learning meaningful and robust features.

To investigate what the model is actually relying on this time, we applied the Grad-Cam algorithm [60] to test images to find highlighted activation areas. This is a method used to see which parts of the image are most influencing the algorithm’s classification. We would expect a classifier relying on true pathologic features to primarily be relying on pixels from the lung fields, whereas a spurious classifier would rely on pixels from regions of the image irrelevant to diagnosis. The results were inconclusive (see Table 5 of the Appendix). Therefore, we cannot affirm whether the model is still relying on shortcuts/confounders to make decisions. This experimental result shows that a model could be adapted to work locally. Still to be shown is that it learns medically relevant features.

## IV. LIMITATIONS OF THE STUDY

In this work, we show with evidence that models created using deep learning which attain high accuracy/AUC on unseen data from seen sources exhibit clear generalization gap issues and are unable to perform as well on data from external unseen sources. Unfortunately, we have too few data sources to conclude definitively that this inconsistency in performance is solely attributed to the differences in data sources or undisclosed preprocessing or other unknown factors. CXRs of the same COVID-19 patient from two different sources would help as would full information on acquisition machines and parameters, which are not available to us at this time. Some of the data sources used in this work underwent partially or fully unknown pre-processing techniques that were not explained by the owners of the datasets. Such missing detail about the data limits our ability to be sure of providing a uniform normalization for all data sources. Due to the rapid and massive growth of the recent literature related to COVID-19 diagnosis using AI methods from X-rays, we cannot be sure that we covered all papers. However, to our knowledge none has proved its ability to generalize to external sites, which is the main focus of this study.

## V. CONCLUSION

In this paper we demonstrate that deep learning models can leverage data-source specific confounders to differentiate between COVID-19 and pneumonia labels. While we eliminated many confounders from earlier work, such as those related to large age discrepancies between populations (pediatric vs adult), image post-processing artifacts introduced by working from low resolution PDF images, and positioning artifacts by pre-segmenting and cropping the lungs, we still saw that deep-learning models were able to learn using data-source specific confounders. Several hypotheses may be considered as to the nature of these confounders. These confounders may be introduced as a result of differences in X-ray procedures as a result of patient clinical severity or patient control procedures. For instance, differences in disease severity may impact patient positioning (standing for ambulatory or emergency department patients vs supine for admitted and ICU patients). In addition, if a particular X-ray machine whose signature is learnable is always used for COVID-19 patients, because it is in a dedicated COVID-19 ward, this would be another method to determine the class in a non-generalizable way.

Using datasets that underwent different pre-processing methods across classes can encourage the model to differentiate classes based on the pre-processing, which is an undesirable outcome. Thus, training the model on a dataset of raw data coming from many sources may provide a general classifier. Even within the same hospital, one must still check to be sure that something approximating what a human would use to differentiate cases is learned.

That being said, using a deep learning classifier trained on positive and negative datasets from the same hospital system, having undergone similar data processing, we were able to train a classifier that performed better than random on chest X-rays from unseen data sources, albeit modestly. Tuning with data from unseen sources provided much improved performance. This suggests that this classification problem may eventually be solvable using deep learning models. However, the theoretical limit of COVID-19 diagnosis, based solely on chest X-ray remains unknown, and consequently also the maximum expected AUC of any machine learning algorithm. Unlike other classification problems that we know can be performed with high accuracy by radiologists, radiologists do not routinely or accurately diagnose COVID-19 by chest X-ray alone. However, an imperfect classifier that has learned features that are not confounders may be combined with other clinical data to create highly accurate classifiers, and as such this area warrants further inquiry.

Our results suggest that, for at least this medical imaging problem, when deep learning is involved it is important to have data from unseen sources (pre-processed in the same way) included in a test set. If there are no unseen sources available, careful investigation is necessary to ensure that what is learned both generalizes and is germane. It points out that future investigation into finding/focusing on features that generalize across sources is quite important. This will enable an evaluation of how helpful CXRs can truly be for COVID-19 diagnosis.

All data and code used in this study are available at https://github.com/kbenahmed89/Pretrained-CNN-For-Covid-19-Prediction-from-Automatically-Lung-ROI-Cropped-X-Rays.

## Figures and Tables

**TABLE 1.** Papers for automatic COVID-19 prediction based on CXR images.

Paper	Performance Result	Testing Dataset
Ozturk et al. [7]	0.99 AUC	train/test split from the same data source
Abbas et al. [8]	0.94 AUC	train/test split from the same data source
Farooq et al. [9]	96.23% Accuracy	train/test split from the same data source
Lv et al. [10]	85.62% Accuracy	train/test split from the same data source
Bassi and Attux [11]	97.80% Recall	train/test split from the same data source
Rahimzadeh and Attar [12]	99.60% Accuracy	train/test split from the same data source
Chowdhury et al. [13]	98.30% Accuracy	train/test split from the same data source
Hemdan et al. [14]	0.89 F1-score	train/test split from the same data source
Karim et al. [15]	83.00% Recall	train/test split from the same data source
Krishnamoorthy et al. [16]	90% Accuracy	train/test split from the same data source
Minaee et al. [17]	90% Specificity	train/test split from the same data source
Afshar et al. [18]	98.3% Accuracy	train/test split from the same data source
Khobahi et al. [19]	93.5% Accuracy	train/test split from the same data source
Moura et al. [20]	90.27% Accuracy	train/test split from the same data source
Kumar et al. [21]	97.7% Accuracy	train/test split from the same data source
Castiglioni et al. [22]	0.80 AUC	train/test split from the same data source
Rahaman et al. [23]	f 89.3% Accuracy	train/test split from the same data source
Hall et al. [24]	0.95 AUC	10-fold cross validation with all sources mixed
Apostolopoulos et al. [25]	92.85% Accuracy	10-fold cross validation with all sources mixed
Apostolopoulos et al. [26]	99.18% Accuracy	10-fold cross validation with all sources mixed
Mukherjee et al. [27]	0.9908 AUC	10-fold cross validation with all sources mixed
Das et al. [28]	1.00 AUC	10-fold cross validation with all sources mixed
Razzak et al. [29]	98.75% Accuracy	10-fold cross validation with all sources mixed
Basu et al. [30]	95.30% Accuracy	5-fold cross validation with all sources mixed
Li et al. [31]	97.01% Accuracy	5-fold cross validation with all sources mixed
Mukherjee et al. [32]	0.9995 AUC	5-fold cross validation with all sources mixed
Moutounet-Cartan et al. [33]	93.9% Accuracy	5-fold cross validation with all sources mixed
Ozturk et al. [34]	98.08% Accuracy	5-fold cross validation with all sources mixed
Khan et al. [35]	89.6% Accuracy	4-fold cross validation with all sources mixed
DeGrave et al. [36]	0.70 AUC	test on an unseen data source
DeGrave et al. [36]	0.995 AUC	train/test split from the same data source
Yeh et al. [37]	40% Specificity	test on an unseen data source
Yeh et al. [37]	80.45% Specificity	train/test split from the same data source
Tartaglione et al. [38]	0.61 AUC	test on an unseen data source
Tartaglione et al. [38]	1.00 AUC	train/test split from the same data source
This work	0.63 AUC	test on an unseen data source
This work	0.96 AUC	train/test split from the same data source

**TABLE 2.** ResNet50 fine-tuned architecture.

Resnet50
Output from base model
Global Average Pooling
Fully Connected (64), ReLU
Dropout=0.5
Batch Normalization
Fully Connected (1), Sigmoid
Trainable parameters: 184K

**TABLE 3.** Performance results of training on a mixture of all data sources and testing on held-out test data from the same sources. Finetune1: Freeze all base model layers, Finetune2: Unfreeze the last 2 convolutional layers.

CNN	Accuracy	Sensitivity	Specificity	AUC
**Resnet50-Finetune1**	**98.1%**	**96.2%**	**98.7%**	**0.997**
Resnet50-Finetune2	97%	95%	97.7%	0.995
VGG-16-Finetune1	88.2%	87.3%	88.4%	0.96
VGG-16-Finetune2	95.5%	93.7%	96%	0.98

**TABLE 4.** Accuracy results of finetuning a model built on multiple sources from both classes to adapt it to work locally. Still to be shown is that it learns medically relevant features.

Class	Data Source	Before	After
COVID-19	BIMCV-COVID-19+ (Spain)	51%	98%
Pneumonia	Chexpert	12%	94%

**TABLE 5.** Grad-Cam visualization of two test samples before and after fine-tuning.

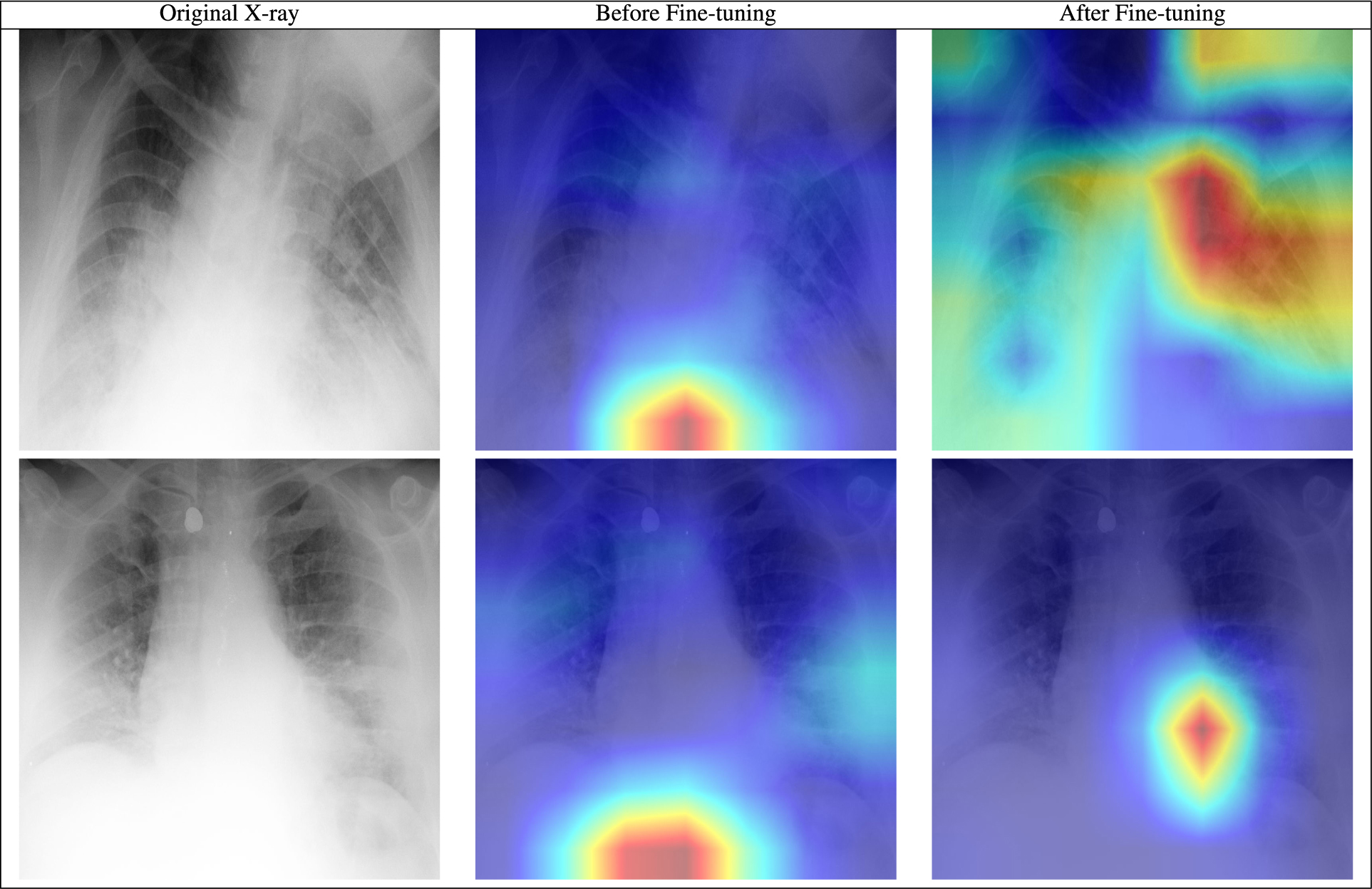

## APPENDIX A

### GRAD-CAM

Table 5 shows Grad-Cam visualization of two test samples before and after fine-tuning the model. As seen in the images, it is hard to confirm that fine-tuning has succeeded in making the model focus on lung area, though the focus there is increased. We do observe that some seemingly random locations outside the lungs are highlighted.

## References

[1] Worldometers, Coronavirus Cases. 12. 22, 2020. [Online]. Available: https://www.worldometers.info/coronavirus/

[2] Weissleder R, Lee H, Ko J, and Pittet MJ, “COVID-19 diagnostics in context,” Sci. Transl. Med, vol. 12, no. 546, 6. 2020, Art. no. eabc1931, doi: 10.1126/scitranslmed.abc1931. 32493791

[3] Wang W, Xu Y, Gao R, Lu R, Han K, Wu G, and Tan W, “Detection of SARS-CoV-2 in different types of clinical specimens,” JAMA, vol. 323, no. 18, pp. 1843–1844, 3. 2020, doi: 10.1001/jama.2020.3786. 32159775 PMC7066521

[4] Eykholt K, Evtimov I, Fernandes E, Li B, Rahmati A, Xiao C, Prakash A, Kohno T, and Song D, “Robust physical-world attacks on deep learning visual classification,” in Proc. IEEE/CVF Conf. Comput. Vis. Pattern Recognit., 6. 2018, pp. 1625–1634.

[5] Chen Y, Jiang G, Li Y, Tang Y, Xu Y, Ding S, Xin Y, and Lu Y, “A survey on artificial intelligence in chest imaging of COVID-19,” BIO Integr., vol. 1, no. 3, pp. 137–146, 12. 2020, doi: 10.15212/bioi-2020-0015.

[6] Alghamdi HS, Amoudi G, Elhag S, Saeedi K, and Nasser J, “Deep learning approaches for detecting COVID-19 from chest X-ray images: A survey,” IEEE Access, vol. 9, pp. 20235–20254, 2021, doi: 10.1109/access.2021.3054484. PMC8545235 34786304

[7] Öztürk CS, Özkaya U, and Barstuğan M, “Classification of Coronavirus (COVID-19) from X-ray and CT images using shrunken features,” Int. J. Imag. Syst. Technol, vol. 31, no. 1, pp. 5–15, 8. 2020, doi: 10.1002/ima.22469. PMC7461473 32904960

[8] Abbas A, Abdelsamea MM, and Gaber MM, “Classification of COVID-19 in chest X-ray images using DeTraC deep convolutional neural network,” Int. J. Speech Technol, vol. 51, no. 2, pp. 854–864, 9. 2020, doi: 10.1007/s10489-020-01829-7. PMC7474514 34764548

[9] Farooq M and Hafeez A, “COVID-ResNet: A deep learning framework for screening of COVID19 from radiographs,” 2020, arXiv:2003.14395. [Online]. Available: http://arxiv.org/abs/2003.14395

[10] Lv D, Qi W, Li Y, Sun L, and Wang Y, “A cascade network for detecting COVID-19 using chest X-rays,” 2020, arXiv:2005.01468. [Online]. Available: http://arxiv.org/abs/2005.01468

[11] Bassi PRAS and Attux R, “A deep convolutional neural network for COVID-19 detection using chest X-rays,” Res. Biomed. Eng., to be published, doi: 10.1007/s42600-021-00132-9.

[12] Rahimzadeh M and Attar A, “A modified deep convolutional neural network for detecting COVID-19 and pneumonia from chest X-ray images based on the concatenation of xception and ResNet50 V2,” Informat. Med. Unlocked, vol. 19, 3. 2020, Art. no. 100360, doi: 10.1016/j.imu.2020.100360. PMC7255267 32501424

[13] Chowdhury MEH, Rahman T, Khandakar A, Mazhar R, Kadir MA, Mahbub ZB, Islam KR, Khan MS, Iqbal A, Emadi NA, Reaz MBI, and Islam MT, “Can AI help in screening viral and COVID-19 pneumonia?” IEEE Access, vol. 8, pp. 132665–132676, 2020, doi: 10.1109/ACCESS.2020.3010287.

[14] El-Din Hemdan E, Shouman MA, and Esmail Karar M, “COVIDX-net: A framework of deep learning classifiers to diagnose COVID-19 in X-ray images,” 2020, arXiv:2003.11055. [Online]. Available: http://arxiv.org/abs/2003.11055

[15] Karim MR, Dohmen T, Cochez M, Beyan O, Rebholz-Schuhmann D, and Decker S, “DeepCOVIDExplainer: Explainable COVID-19 diagnosis from chest X-ray images,” in Proc. IEEE Int. Conf. Bioinf. Biomed. (BIBM), 12. 2020, pp. 1034–1037, doi: 10.1109/bibm49941.2020.9313304.

[16] Krishnamoorthy S, Ramakrishnan S, Colaco LB, Dias A, Gopi IK, Gowda GAG, and Aishwarya KC, “Comparing a deep learning model’s diagnostic performance to that of radiologists to detect Covid-19 features on chest radiographs,” Indian J. Radiol. Imag, vol. 31, no. 5, p. 53, 2021, doi: 10.4103/ijri.IJRI_914_20. PMC7996677 33814762

[17] Minaee S, Kafieh R, Sonka M, Yazdani S, and Jamalipour Soufi G, “Deep-COVID: Predicting COVID-19 from chest X-ray images using deep transfer learning,” Med. Image Anal, vol. 65, 10. 2020, Art. no. 101794, doi: 10.1016/j.media.2020.101794. 32781377 PMC7372265

[18] Afshar P, Heidarian S, Naderkhani F, Oikonomou A, Plataniotis KN, and Mohammadi A, “COVID-CAPS: A capsule network-based framework for identification of COVID-19 cases from X-ray images,” Pattern Recognit. Lett, vol. 138, pp. 638–643, 10. 2020, doi: 10.1016/j.patrec.2020.09.010. 32958971 PMC7493761

[19] Khobahi S, Agarwal C, and Soltanalian M, “Coronet: A deep network architecture for semi-supervised task-based identification of COVID-19 from chest X-ray images,” MedRxiv, doi: 10.1101/2020.04.14.20065722.

[20] De Moura J, Garcia LR, Vidal PFL, Cruz M, Lopez LA, Lopez EC, Novo J, and Ortega M, “Deep convolutional approaches for the analysis of COVID-19 using chest X-ray images from portable devices,” IEEE Access, vol. 8, pp. 195594–195607, 2020, doi: 10.1109/access.2020.3033762. PMC8545263 34786295

[21] Kumar R, Arora R, Bansal V, Sahayasheela VJ, Buckchash H, Imran J, Narayanan N, Pandian GN, and Raman B, “Accurate prediction of COVID-19 using chest X-ray images through deep feature learning model with SMOTE and machine learning classifiers,” MedRxiv, to be published, doi: 10.1101/2020.04.13.20063461.

[22] Castiglioni I, Ippolito D, Interlenghi M, Monti CB, Salvatore C, Schiaffino S, Polidori A, Gandola D, Messa C, and Sardanelli F, “Artificial intelligence applied on chest X-ray can aid in the diagnosis of COVID-19 infection: A first experience from Lombardy, Italy,” MedRxiv, to be published, doi: 10.1101/2020.04.08.20040907. PMC7850902 33527198

[23] Rahaman MM, Li C, Yao Y, Kulwa F, Rahman MA, Wang Q, Qi S, Kong F, Zhu X, and Zhao X, “Identification of COVID-19 samples from chest X-ray images using deep learning: A comparison of transfer learning approaches,” J. X-Ray Sci. Technol, vol. 28, no. 5, pp. 821–839, 9. 2020, doi: 10.3233/xst-200715. PMC7592691 32773400

[24] Hall LO, Paul R, Goldgof DB, and Goldgof GM, “Finding covid-19 from chest X-rays using deep learning on a small dataset,” 2020, arXiv:2004.02060. [Online]. Available: http://arxiv.org/abs/2004.02060

[25] Apostolopoulos ID and Mpesiana TA, “Covid-19: Automatic detection from X-ray images utilizing transfer learning with convolutional neural networks,” Phys. Eng. Sci. Med, vol. 43, no. 2, pp. 635–640, 6. 2020, doi: 10.1007/s13246-020-00865-4. 32524445 PMC7118364

[26] Apostolopoulos ID, Aznaouridis SI, and Tzani MA, “Extracting possibly representative COVID-19 biomarkers from X-ray images with deep learning approach and image data related to pulmonary diseases,” J. Med. Biol. Eng, vol. 40, no. 3, pp. 462–469, 5 2020, doi: 10.1007/s40846-020-00529-4. PMC7221329 32412551

[27] Mukherjee H, Ghosh S, Dhar A, Obaidullah SM, Santosh KC, and Roy K, “Deep neural network to detect COVID-19: One architecture for both CT scans and chest X-rays,” Int. J. Speech Technol, vol. 51, no. 5, pp. 2777–2789, 5 2021, doi: 10.1007/s10489-020-01943-6. PMC7646727 34764562

[28] Das D, Santosh KC, and Pal U, “Truncated inception net: COVID-19 outbreak screening using chest X-rays,” Phys. Eng. Sci. Med, vol. 43, no. 3, pp. 915–925, 6. 2020, doi: 10.1007/s13246-020-00888-x. 32588200 PMC7315909

[29] Rehman A, Naz S, Khan A, Zaib A, and Razzak I, “Improving coronavirus (COVID-19) diagnosis using deep transfer learning,” MedRxiv, to be published, doi: 10.1101/2020.04.11.20054643.

[30] Basu S, Mitra S, and Saha N, “Deep learning for screening COVID-19 using chest X-ray images,” in Proc. IEEE Symp. Ser. Comput. Intell. (SSCI), 12. 2020, pp. 2521–2527.

[31] Li T, Han Z, Wei B, Zheng Y, Hong Y, and Cong J, “Robust screening of COVID-19 from chest X-ray via discriminative cost-sensitive learning,” 2020, arXiv:2004.12592. [Online]. Available: http://arxiv.org/abs/2004.12592

[32] Mukherjee H, Ghosh S, Dhar A, Obaidullah SM, Santosh KC, and Roy K, “Shallow convolutional neural network for COVID-19 outbreak screening using chest X-rays,” Cognit. Comput, to be published, doi: 10.1007/s12559-020-09775-9. PMC7863062 33564340

[33] Moutounet-Cartan PGB, “Deep convolutional neural networks to diagnose COVID-19 and other pneumonia diseases from posteroanterior chest X-rays,” 2020, arXiv:2005.00845. [Online]. Available: http://arxiv.org/abs/2005.00845

[34] Ozturk T, Talo M, Yildirim EA, Baloglu UB, Yildirim O, and Rajendra Acharya U, “Automated detection of COVID-19 cases using deep neural networks with X-ray images,” Comput. Biol. Med, vol. 121, 6. 2020, Art. no. 103792, doi: 10.1016/j.compbiomed.2020.103792. 32568675 PMC7187882

[35] Khan AI, Shah JL, and Bhat MM, “CoroNet: A deep neural network for detection and diagnosis of COVID-19 from chest X-ray images,” Comput. Methods Programs Biomed, vol. 196, 11. 2020, Art. no. 105581, doi: 10.1016/j.cmpb.2020.105581. 32534344 PMC7274128

[36] DeGrave AJ, Janizek JD, and Lee S-I, “AI for radiographic COVID-19 detection selects shortcuts over signal,” medRxiv, to be published, doi: 10.1101/2020.09.13.20193565.

[37] Yeh C-F, “A cascaded learning strategy for robust COVID-19 pneumonia chest X-ray screening,” 2020, arXiv:2004.12786. [Online]. Available: http://arxiv.org/abs/2004.12786

[38] Tartaglione E, Barbano CA, Berzovini C, Calandri M, and Grangetto M, “Unveiling COVID-19 from CHEST X-ray with deep learning: A hurdles race with small data,” Int. J. Environ. Res. Public Health, vol. 17, no. 18, p. 6933, 9. 2020, doi: 10.3390/ijerph17186933. PMC7557723 32971995

[39] Panwar H, Gupta PK, Siddiqui MK, Morales-Menendez R, and Singh V, “Application of deep learning for fast detection of COVID-19 in X-rays using nCOVnet,” Chaos, Solitons Fractals, vol. 138, 9. 2020, Art. no. 109944, doi: 10.1016/j.chaos.2020.109944. 32536759 PMC7254021

[40] Hosseinzadeh Kassani S, Hosseinzadeh Kassasni P, Wesolowski MJ, Schneider KA, and Deters R, “Automatic detection of coronavirus disease (COVID-19) in X-ray and CT images: A machine learning-based approach,” 2020, arXiv:2004.10641. [Online]. Available: http://arxiv.org/abs/2004.10641 10.1016/j.bbe.2021.05.013 PMC8179118 34108787

[41] Oh Y, Park S, and Ye JC, “Deep learning COVID-19 features on CXR using limited training data sets,” IEEE Trans. Med. Imag, vol. 39, no. 8, pp. 2688–2700, 8. 2020, doi: 10.1109/tmi.2020.2993291. 32396075

[42] Wang L, Lin ZQ, and Wong A, “COVID-net: A tailored deep convolutional neural network design for detection of COVID-19 cases from chest X-ray images,” Sci. Rep, vol. 10, no. 1, p. 19549, 11. 2020, doi: 10.1038/s41598-020-76550-z. 33177550 PMC7658227

[43] Asif S and Amjad K, “Automatic COVID-19 detection from chest radiographic images using convolutional neural network,” medRxiv, to be published, doi: 10.1101/2020.11.08.20228080.

[44] Rajaraman S and Antani S, “Training deep learning algorithms with weakly labeled pneumonia chest X-ray data for COVID-19 detection,” medRxiv, to be published, doi: 10.1101/2020.05.04.20090803.

[45] Cherezov D, Paul R, Fetisov N, Gillies RJ, Schabath MB, Goldgof DB, and Hall LO, “Lung nodule sizes are encoded when scaling CT image for CNN’s,” Tomography, vol. 6, no. 2, pp. 209–215, 6. 2020, doi: 10.18383/j.tom.2019.00024. 32548298 PMC7289250

[46] Roberts M, AIX-COVNET, Driggs D, Thorpe M, Gilbey J, Yeung M, Ursprung S, Aviles-Rivero AI, Etmann C, McCague C, Beer L, Weir-McCall JR, Teng Z, Gkrania-Klotsas E, Rudd JHF, Sala E, and Schönlieb C-B, “Common pitfalls and recommendations for using machine learning to detect and prognosticate for COVID-19 using chest radiographs and CT scans,” Nature Mach. Intell, vol. 3, no. 3, pp. 199–217, 3. 2021, doi: 10.1038/s42256-021-00307-0.

[47] Cohen J. Paul, Morrison P, and Dao L, “COVID-19 image data collection,” 2020, arXiv:2003.11597. [Online]. Available: http://arxiv.org/abs/2003.11597

[48] Radiopaedia. Radiopaedia Blog RSS. 12. 10, 2020. [Online]. Available: https://radiopaedia.org/

[49] SIRM, COVID-19 DATABASE | SIRM. Accessed: Feb. 20, 2021. [Online]. Available: https://www.sirm.org/category/senza-categoria/covid-19/

[50] de la Iglesia Vayá M, Saborit JM, Montell JA, Pertusa A, Bustos A, Cazorla M, Galant J, Barber X, Orozco-Beltrán D, García-García F, Caparrós M, González G, and Salinas JM, “BIMCV COVID-19+: A large annotated dataset of RX and CT images from COVID-19 patients,” 2020, arXiv:2006.01174. [Online]. Available: https://arxiv.org/abs/2006.01174

[51] Desai S, Baghal A, Wongsurawat T, Al-Shukri S, Gates K, Farmer P, Rutherford M, Blake G, Nolan T, Powell T, Sexton K, Bennett W, and Prior F, “Chest imaging with clinical and genomic correlates representing a rural COVID-19 positive population [data set],” Cancer Imag. Arch., New York, NY, USA, Tech. Rep, 2020, doi: 10.7937/tcia.2020.py71-5978. PMC7686304 33235265

[52] Winther HB, Laser H, Gerbel S, Maschke SK, Hinrichs JB, Vogel-Claussen J, Wacker FK, Höper MM, and Meyer BC, “COVID-19 image repository,” Hannover Med. School, Inst. Diagnostic Interventional Radiol., Hannover, Germany, Tech. Rep, 2020, doi: 10.6084/m9.figshare.12275009.v1.

[53] Wang X, Peng Y, Lu L, Lu Z, Bagheri M, and Summers RM, “ChestX-ray8: hospital-scale chest X-ray database and benchmarks on weakly-supervised classification and localization of common thorax diseases,” in Proc. IEEE Conf. Comput. Vis. Pattern Recognit. (CVPR), 7. 2017, pp. 2097–2106.

[54] Irvin J, Rajpurkar P, Ko M, and Yu Y, “Chexpert: A large chest radiograph dataset with uncertainty labels and expert comparison,” in Proc. AAAI Conf. Artif. Intell., vol. 33, 2019, pp. 590–597.

[55] Bustos A, Pertusa A, Salinas J-M, and de la Iglesia-Vayá M, “Pad-Chest: A large chest X-ray image dataset with multi-label annotated reports,” Med. Image Anal, vol. 66, 12. 2020, Art. no. 101797, doi: 10.1016/j.media.2020.101797. 32877839

[56] Rajaraman S, Siegelman J, Alderson PO, Folio LS, Folio LR, and Antani SK, “Iteratively pruned deep learning ensembles for COVID-19 detection in chest X-rays,” IEEE Access, vol. 8, pp. 115041–115050, 2020, doi: 10.1109/access.2020.3003810. 32742893 PMC7394290

[57] He K, Zhang X, Ren S, and Sun J, “Deep residual learning for image recognition,” in Proc. IEEE Conf. Comput. Vis. Pattern Recognit. (CVPR), 6. 2016, pp. 770–778.

[58] Kingma DP and Ba J, “Adam: A method for stochastic optimization,” 2014, arXiv:1412.6980. [Online]. Available: http://arxiv.org/abs/1412.6980

[59] McNemar Q, “Note on the sampling error of the difference between correlated proportions or percentages,” Psychometrika, vol. 12, no. 2, pp. 153–157, 6. 1947, doi: 10.1007/bf02295996. 20254758

[60] Selvaraju RR, Cogswell M, Das A, Vedantam R, Parikh D, and Batra D, “Grad-CAM: Visual explanations from deep networks via gradient-based localization,” in Proc. IEEE Int. Conf. Comput. Vis. (ICCV), 10. 2017, pp. 618–626.

